# Audit of antipsychotic prescribing and monitoring for the management of behavioural and psychological symptoms of dementia

**DOI:** 10.1192/bjo.2021.320

**Published:** 2021-06-18

**Authors:** Catrin Thomas, Sharmi Bhattacharyya, Elizabeth Bond

**Affiliations:** 1 ST4 Old Age Psychiatry, Betsi Cadwaladr University Health Board; 2 Consultant Old Age Psychiatrist and Clinical Lead, Betsi Cadwaladr University Health Board, Visiting Professor, University of Chester; 3 Head of Pharmacy, Mental Health and Learning Disabilities Division, Betsi Cadwaladr University Health Board

## Abstract

**Aims:**

To assess the use of a piloted shorter version of the local Checklist for Antipsychotic Initiation and Review (CAIR) form by an Older Persons Community Mental Health Team (OPCMHT), and to assess whether the National Institute for Health and Care Excellence (NICE) guideline on use of antipsychotics for the management of behavioural and psychological symptoms of dementia (BPSD) is being adhered to.

**Method:**

Retrospective audit analysing notes of all patients currently open to the OPCMHT that are prescribed an antipsychotic medication for the management of BPSD. Patients with a diagnosis of any subtype of dementia and prescribed any antipsychotic were included. Data collected from paper notes using an audit proforma.

**Result:**

The total number of patients was 11. The most common diagnosis was Alzheimer's disease (45%), followed by mixed type dementia (36%), vascular dementia (9%) and Lewy Body dementia (9%). The majority of the patients reside in their own home (64%) whilst the remaining 36% reside in a residential home for the elderly and mentally infirm. The CAIR form was present in 73% of the patient's notes, however only 37% had the new, piloted, shorter version of the CAIR form. Of the CAIR forms present, only 63% were fully completed. There was documented evidence that 100% of patients had an assessment of underlying causes of their challenging behaviour; that non-pharmacological interventions were tried first; and that target symptoms were identified. There was evidence of a discussion with the patient or carer about the risks and benefits of antipsychotic use for all patients, however the details of the discussion was often vague. All patients had a review of the antipsychotic medication within the last three months.

**Conclusion:**

There was evidence that pre-prescribing assessments are being undertaken for all patients. There needs to be clearer documentation of the discussions had with patients and carers about the risks and benefits of using antipsychotic medications for management of BPSD. A teaching session was held at the team meeting to highlight the risks and benefits. The team will ensure that they provide a health board approved leaflet to each patient and carer following their discussion. Only 73% of the patients had a CAIR form in their notes and the team favour the original version. The team will revert back to using the original version of the CAIR form as it has more space allocated to document ongoing reviews. We will re-audit in 6 months time.

